# The Armour Skeleton

**Published:** 1887-08-27

**Authors:** W. E. Fothergill


### The Armour Skeleton.

By W. E. Fothergill.

Egotistical man has a habit of using all words in a narrow and personal sense. He calmly states that a thing is "pleasant" because he happens to like it, or "disagreeable"—perhaps he uses a stronger word—when it fails to catch his taste or humour; and by employing them in a variety of uses coinciding exactly with the number of human beings who have existed on this planet he has made "good" and "evil" comparative and not ultimate terms. Acting on this principle he has given to the word "skeleton" such a selfish meaning that he applies it solely to that airy and grinning frame which sometimes confronts him in anatomical museums, or whose general appearance he sees inaccurately represented on the older tombstones in country churchyards. Except in moments of conscious care and strictness he does not bestow the title of skeleton upon the framework of even his humbler fellow-vertebrates, and to the invertebrates he refuses it altogether, looking down upon them from the height of his backboned superiority.

Yet he and they are fundamentally the same. All animals consist of certain soft tissues supported and protected by the arrangements known as skeletons: which arrangements may be produced and carried inside the softer parts, in which case they are called backboned skeletons; or may be formed by the skin and worn outside, when they are entitled "armour skeletons."

The desire for a protection, for a covering of some sort, is plainly seen in all life's children; after food, raiment is the natural sequence of demand. The chalk-forming animalcule makes itself a coat of lime; many of its brothers use flint instead, and use it to good and decorative purpose. The Holothuria for example, trims its gown with a delicate and admirably arranged pattern of intertwined anchors and rings. The sponges employ the same materials. The coral polyp's life-work is to build a home for itself upon the ruins of those that housed its fathers; rearing a pile which ultimately provides a resting-place for the South Sea islander and a profitable occupation for the lapidary. Arenicola and many a kindred worm protects itself by boring a sheltering hole in the mud of ages; the more aspiring Aphrodite mats her fair hair across her back and forms a thickly felted coat. Talking of worms, I recall a certain worm-wise and worm-loving old German professor who named a quintet of lovely daughters after the creatures to whom his life's labour was devoted—Aphrodite, Polymia, Eunice, Nereis, and Tomoptera—getting less euphonious as he came to the last fair maid, who, one cannot help hoping, was enough of a hoyden to permit of her name being contracted into "Tommy."

Rising in life's scale, the oysters clothe themselves in pearly shell, and their cousins the snails use a similar covering. But it is in the animals with jointed limbs that we find the armour skeleton in perfection. All the great host of insects wear light, strong plate-armour, beautifully jointed to suit the action of their dainty limbs; but our point will best appear if we cast a glance at the crabs and their kin. We have seen what varied steps backboneless nature took before she completed the epicure's environment, and produced the consummate lobster. His living skeleton lines his mouth and throat—the lobster's, not the epicure's—and forms a curious mill of teeth in his stomach; it covers his head and chest with one great shield, and divides itself into a multitude of plates to protect his body and his twenty pairs of limbs. The youthful lobster who wishes to grow naturally finds some difficulty in stretching his skin. He solves the problem by loosening it all over, and cracking it round the middle. Then he spits out the bones that lined his stomach, pulls his head out of one end of the shell and his tail out of the other, and crawls away into obscurity till he can grow a new garment of larger dimensions. As lobsters go on growing all their lives, the process has to be repeated pretty frequently, which makes the armour skeleton a little inconvenient. Early in the history of the world, when the lobster family was the reigning one, there were gigantic lobsters, twelve feet in length, with living armour like the plates of an ironclad. Why have we none such now, but sigh almost in vain for enough to flavour our salad ?

Think of all the animals, now existing, that have had outer parts serving to protect them from their enemies, and giving fixed points for their muscles to act on : all are small and insignificant. The cause is plain. The armour skeleton, if strong enough to protect a large animal, must be so heavy and clumsy as to cause more inconvenience than it is worth. The forms of life which trusted to it in past ages have perished in the struggle for existence ; and all that we have to remind us of the great crustaceans are a few fossils and some insignificant descendants.

What then enabled the birds, beasts, and fishes to gain so signal a victory over their armour-plated rivals in air, and earth, and sea ? To put it briefly—brains and nerves. The backboned animal has been defined as a nervous system with a skeleton to support it and arrangements for its nutrition. The creatures that were to be the ancestors of the low backboned forms did not observe the awkwardness of their outside skeletons till they got nervous enough to see and hear and feel things that made them want to move quickly. So the plate-armour was given up only when the animal had good eyes, and ears, and nerves of feeling, with a good brain, and nerves of motion which enabled it to see danger quickly and to avoid it.

But these nerves needed some protection, and since speed was to replace thickness of skin, the muscles required something firm to work upon. So the backbone came into use, with a canal for the spinal nerve inside, and a brain-box at one end. From the simple rod of cartilage in the lowest backboned animals the whole inside skeleton of the higher ones was formed ; and from these descended the whole great vertebrate kingdom, including that highest vertebrate, who, as I hinted at the beginning of this paper, cherishes an aristocratic contempt for his poor relations.

A point worthy of note is that the cuttle-fish, which is the descendant of a fore-seeing member of the snail and oyster family, has actually gained many of the vertebrate's advantages by reducing its shell to a rod of bone quite inside its back. The result of this shrewd action is that the cuttle-fishes are now the largest and most formidable of invertebrate animals.

But how strong in nature is the desire for protection ! how weak is faith in one's own powers and in providence ! In every class of vertebrate animals we find some greedy members, who, not content with spine and nerves, have further assumed some outward protection, returning again to their wallowing in a metaphorical mire of scales and spikes, of tortoise-shell and prickles.

The punishment has always been the complete extermination of the race, or its reduction to a few wretched members, skulking in the darkness of night in a few holes and corners of the earth and sea.

A race of fishes with heavy plates of bone-thickened skin, once the rulers of the world of waters, has now only a few remaining species. Amongst the reptiles we have the most successful armour-bearers of the present day. The scaly snakes, however, have to perform the lobster-trick once or twice a year, and often perish in the attempt. The crocodiles are still a large and lively tribe, but their present condition is insignificance itself compared with their former supremacy. The turtles are a fairly active family, yet mock-turtle is by no means rare. Even the nature of the common tortoise is not too well known. "Have you got a dog-ticket for that creature ?" asked the ticket-collector of a passenger who had a tortoise on the seat beside him in a railway carriage. "No," was the answer, "I don't need a ticket ; this ain't a dog." The collector retired, consulted the station-master, studied the rules of the company regarding the transit of animals, and having satisfied his mind, returned and delivered the following interesting lecture on natural history : "You're quite right, sir, 'tain't a dog ; cats is dogs, rabbits is dogs, but a tortoise is a hinsect."

The birds, types of faith and freedom, have, to their praise be it said, attempted no return to outward protection since the time when they were hardly distinguishable from the reptiles, from which they are descended. Armadilloes, porcupines, and hedgehogs are amongst the few faithless mammals still remaining who bear scales and prickles. The hugely thickened skins of the hippopotami and rhinoceri are a move in the same retrograde direction, so the decline and fall of the pachydermata will probably form a sad but interesting chapter in the natural history of the future.

Man himself, from the time when he became a worker in brass and iron, adopted an armour skeleton ; but with the refinement and advantage that he did not wear it when he thought he was not likely to want it. But as brains again assumed the mastery over bones, breastplate and greaves were gradually but completely replaced by doublet and hose ; and now men do not even wall their towns.

It seems, then, that in every sense we must wear our bones inside our flesh and not outside ; and preserve a soft, sensitive, yielding exterior. May not the rule apply to minds as well as bodies ; and readiness to receive and act upon impressions be a better mental protection than the case-hardening which sensitive souls so often choose to adopt ? Carrying the idea further still, a student of the comparative anatomy of religions might, if he were fond of analogies, for once draw a parallel that would not lead him far astray. Suppose he argued thus :—

All religions were devoid of backbone till Christ gave morals motive force by bearing witness with His death to the doctrine of universal love. Since then the Church, in manner worthy of an adolescent lobster, has passed through a long series of vertebrate forms, crawling out of one shell of rites and ceremonies only to make another very little larger. It is only now that men are developing faith enough to keep their moral bones inside their souls, and present to one another no armour of inflexible dogma, but only the warm flesh and blood of human kindness.

